# Previous Radiotherapy Increases the Efficacy of IL-2 in Malignant Pleural Effusion: Potential Evidence of a Radio-Memory Effect?

**DOI:** 10.3389/fimmu.2018.02916

**Published:** 2018-12-11

**Authors:** Dawei Chen, Xinyu Song, Haiyong Wang, Zhenwu Gao, Wenjuan Meng, Shuquan Chen, Yunfeng Ma, Youda Wang, Kong Li, Jinming Yu, Jinbo Yue

**Affiliations:** ^1^Department of Radiation Oncology, Shandong Cancer Hospital affiliated to Shandong University, Jinan, China; ^2^Department of Internal Medicine-Oncology, Shandong Cancer Hospital affiliated to Shandong University, Jinan, China; ^3^School of Medicine and Life Sciences, University of Jinan-Shandong Academy of Medical Sciences, Jinan, China; ^4^Department of oncology, Affiliated Hospital of Weifang Medical University, Weifang, China; ^5^Weifang People's Hospital, Weifang, China; ^6^Laiwu Hospital of Traditional Chinese Medicine, Laiwu, China; ^7^Laiwu People's Hospital, Laiwu, China; ^8^Linyi City People's Hospital, Linyi, China

**Keywords:** non-small-cell lung cancer (NSCLC), radiotherapy, radio-memory effect, immunotherapy, interleukin-2 (IL-2), malignant pleural effusion (MPE)

## Abstract

Preclinical and clinical studies have shown that prior receipt of radiotherapy enhances antitumor immune responses, a phenomenon we call the “radio-memory effect.” However, all of the evidence regarding this effect to date comes from work with PD1/PDL1 inhibitors. Here we explored whether this effect also occurs with other forms of immune therapy, specifically interleukin-2 (IL-2). We retrospectively assessed outcomes in patients with malignant pleural effusion (MPE) who had previously received radiotherapy for non-small-cell lung cancer (NSCLC) within 18 months before the intrapleural infusion of IL-2 or cisplatin. Radiotherapy sites included lungs, thoracic lymph nodes, and intracranial. All patients received intrapleural infusion of IL-2 or cisplatin, and most had had several cycles of standard chemotherapy for NSCLC. We identified 3,747 patients with MPE (median age 64 years [range 29–88)) treated at one of several institutions from August 2009 through February 2015; 642 patients had been treated with IL-2 and 1102 with cisplatin and had survived for at least 6 months afterward. Among those who received IL-2, 288 had no radiotherapy, 324 had extracranial (i.e., thoracic) radiotherapy, and 36 had intracranial radiotherapy. The median follow-up time for surviving patients was 38 months. Patients who had received extracranial radiotherapy followed by IL-2 had significantly longer PFS than patients who had not received extracranial radiotherapy (i.e., either no radiotherapy or intracranial radiotherapy). Patients who had received intracranial or extracranial radiotherapy followed by IL-2 had significantly longer OS than did other patients. No survival advantage was noted for prior radiotherapy among patients who received intrapleural cisplatin. We speculate that previous radiotherapy could enhance the efficacy of subsequent intrapleural infusion of IL-2, a “radio-memory” effect that could be beneficial in future studies.

## Introduction

Immune checkpoint inhibitors have recently gained popularity owing to their efficacy and low toxicity (1). Immunotherapy, in combination with conventional oncology treatment, can activate the body's autoimmune response to recognize tumor cells throughout the body (2). Radiotherapy has classically been considered a form of local treatment, causing direct damage to DNA in tumor cells. Because some immune cells are inherently sensitive to radiotherapy, radiotherapy also has both immunosuppressive and immunostimulatory activities (3). Combinations of local radiotherapy and systemic immunotherapy have been shown recently to have significant advantages in preclinical and clinical studies (4). Some studies have also confirmed that radiotherapy combined with immunotherapy can produce abscopal effects and perhaps what we term a “radio-memory” effect such that the addition of one to the other has synergistic effects (5, 6). Although radiation-induced abscopal effects have been reported in many studies (7–10), little is known about the radio-memory effect.

In terms of immunostimulatory effects, localized radiotherapy can stimulate systemic immune responses (3) by promoting the expression of tumor-associated antigens and the production of new tumor antigens to activate antitumor immune responses, counteracting the tumor's ability to inhibit antigen presentation. For instance, MHC-1, a key antigen recognized by CD8+ T cells, is significantly reduced in tumor cells (11). Radiotherapy can effectively promote the expression of MHC-1, promote the maturation of dendritic cells, and infiltrate tumors (12); reduce the numbers of Tregulatory cells (Tregs) in tumors; expand T-cell lineages; and enhance T cell migration. Radiotherapy has also been shown to partially or in some cases completely convert non-immunogenic tumors into immunogenic tumors (13). Radiotherapy in combination with anti-PD1/PDL1 antibodies, anti-CTLA4 antibodies, immune cytokines, dendritic cell vaccines, and Toll-like receptor antagonists can control local tumor progression, thereby improving overall survival (OS) and inducing specific immune responses to cancer (14).

The KEYNOTE-001 and PACIFIC studies confirmed that having had radiotherapy followed by treatment with a PD1/PDL1 inhibitor could produce a memory-specific immune anti-cancer effect (14, 15). We postulate that this result results from radiotherapy acting as a catalyst or ignition agent that may change a patient's overall immune microenvironment; radiotherapy may enhance the production and storage of immune memory cells, which when followed by immunotherapy would amplify the efficacy of the immunotherapy. With the above hypothesis, we evaluated the effects of intrapleural infusion of IL-2 for the treatment of malignant pleural effusions (MPE) caused by lung cancer, particularly whether these effects varied in patients who had received radiotherapy vs. in those who had not. Specifically, we sought to determine whether the radio-memory effect could occur after radiotherapy combined with other immunotherapy agents, or whether this effect is restricted to anti-PD1/PDL1 drugs.

## Methods

### Patients

We retrospectively identified and reviewed records of patients with MPE to identify patients with non-small-cell lung cancer (NSCLC) who had received intrapleural infusion of IL-2 or cisplatin, with or without prior radiotherapy. Patients were aged 18 years or older, had adequate organ function, with no history of pneumonitis, systemic immunosuppressive therapy, or other autoallergic diseases. Efficacy evalution and disease progression were determined with the Response Evaluation Criteria in Solid Tumors (RECIST) version 1.1. All patients provided written informed consent, and this study was approved by the appropriate institutional review boards. All patients enrolled in our study were treated at one of the following institutions: Shandong Cancer Hospital and Institute; Affiliated Hospital of Weifang Medical University; Weifang People's Hospital; Laiwu Chinese Medicine Hospital; and Linyi People's Hospital, all in Shandong, China.

### Procedures

Patients received intrapleural infusion of one cycle of either IL-2 (20,000 U) or cisplatin alone (40–60 mg) until the effusion progressed. Before this infusion, all patients underwent more than one ultrasound-guided pleural catheterization for drainage to remove as much of the pleural effusion as possible. To ensure the uniform distribution of the agents in the pleural cavity, patients were advised to turn over smoothly every 15 min.

Patients were considered to have a history of radiotherapy if they had received any radiotherapy for NSCLC at any time during the 18 months before the intrapleural treatment. Radiotherapy sites included the lungs or metastases in the thoracic lymph nodes, brain, and other extracranial metastatic sites. Radiotherapy schedules before MPE were categorized as (1) chemotherapy followed by concurrent chemoradiotherapy, (2) upfront concurrent chemoradiotherapy followed by adjuvant chemotherapy, (3) induction chemotherapy followed by sequential radiotherapy, concurrent (4) chemoradiotherapy only, or (5) intracranial radiotherapy. Radiotherapy type was categorized as conventional (i.e., 2-dimensional), three-dimensional conformal radiotherapy, or intensity-modulated radiation therapy.

### Data Collection and Evaluation Criteria

Clinicopathologic data collected for all patients included sex, age, Eastern Cooperative Oncology Group (ECOG) performance status score, smoking history. Hematologic indicator including total lymphocytic counts, neutrophils and neutrophil–lymphocyte Ratio (NLR) were also collected. Response to treatment (by RECIST v1.1) were evaluated as described elsewhere (7, 15, 17). Short-term efficacy was classified as complete response (CR; MPE and symptoms disappeared and the patient's condition was stable for >8 weeks), partial response (PR; MPE size reduced by 50%, symptoms improved, and no subsequent growth in the MPE over 8 weeks), stable disease (SD; MPE size reduced by < 50% or unchanged), or progressive disease (PD; MPE size increased). The objective response rate (ORR) included both CR and PR, and the disease (MPE) control rate (DCR) included CR, PR, and SD.

### Outcomes

The primary objective was to determine whether prior receipt of radiotherapy affected PFS or OS after intrapleural IL-2 or cisplatin treatment. The secondary objective was to determine the effect of previous extracranial radiotherapy on PFS and OS, to account for potential blood-brain barrier effects. Additional objectives included evaluating the effect of previous radiotherapy on treatment efficacy and pulmonary toxicity. Progression-free survival (PFS) was defined as the interval between the initiation of intrapleural IL-2 (or cisplatin) and the time to either effusion progression or death. Overall survival (OS) was measured from the date of the initiation of intrapleural IL-2 to the date of death from any cause or the last known follow-up date.

### Statistical Analysis

All statistical analyses were done with SPSS (Statistical Package for the Social Sciences) version 17.0 and Prism GraphPad 6.0. Clinicopathologic characteristics and short-term efficacy were analyzed by using χ^2^ tests, Fisher's exact tests, or Student's *t* tests. Independent predictors associated with PFS and OS were identified by using a Cox regression model. PFS and OS were analyzed with the Kaplan-Meier method, with differences between groups evaluated with log-rank tests. Two-sided *P-*values of < 0.05 were considered statistically significant.

## Results

### Patient Characteristics

**Figure 1:**
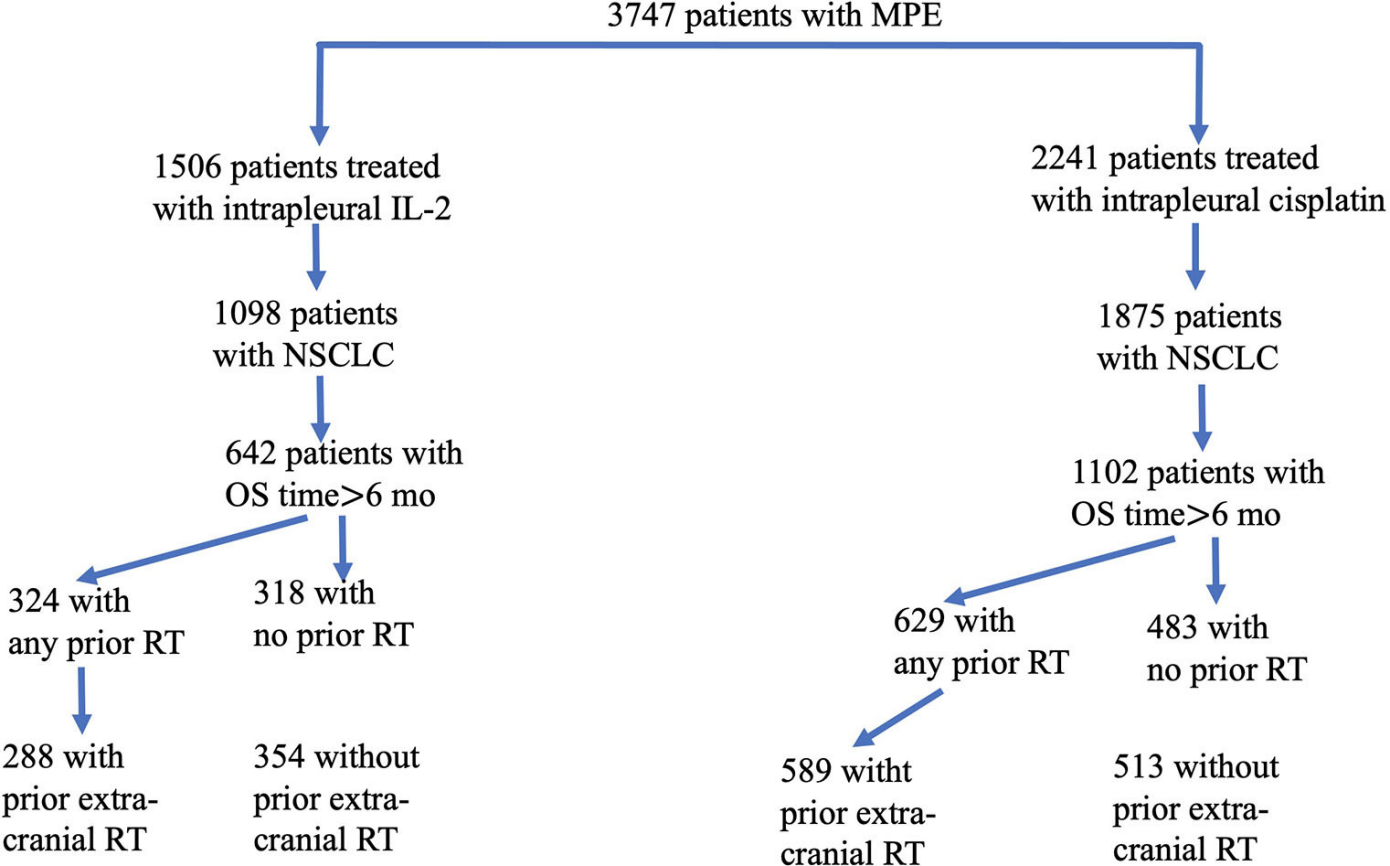
Flowchart of eligible patients enrolled in this study. From a total of 3,747 patients with malignant pleural effusion (MPE), we identified 1,506 who had been treated with interpleural interleukin-2 (IL-2) and 2,241 who had been treated with intrapleural cisplatin. Of the 1,098 patients given IL-2 (and the 1,875 patients given cisplatin) who had non-small cell lung cancer, 642 who had received IL-2 survived for more than 6 months, and 1,102 who had received cisplatin survived for more than 6 months. Patients in each group were subdivided according to whether they had any vs. no radiotherapy (RT), or extracranial vs. no extracranial RT.

A total of 3,747 patients with MPE were treated at the participating hospitals between August 2009 and February 2015. Of these patients, 1,506 received intrapleural IL-2 and the other 2,241 received intrapleural cisplatin. Among the 1,506 patients given IL-2, 1,098 had NSCLC; after exclusion of 456 patients who survived for < 6 months after treatment, the study group consisted of 642 patients (Figure 1). The control group (i.e., those given intrapleural cisplatin but not IL-2) consisted of 1,102 patients with NSCLC who survived for more than 6 months after treatment. The enrollment flowchart is shown in Figure 1, and baseline patient characteristics are shown in Table 1. The median age of the 642 patients in the study group was 62 years (range 29–87). Most patients had received intrapleural chemotherapy and several lines of systemic therapy before receiving the intrapleural IL-2. Slightly more than half of the 642 patients given IL-2 (324, or 50.5%) had previously received radiotherapy, which was extracranial in 288 (44.8%). The median follow-up for patients alive at this analysis was 38 months. Radiotherapy was delivered for a median period of 6.4 months (range 0.8–85.0, IQR 3.0–15.5), before the first cycle of intrapleural IL-2.

**Table 1:** Baseline characteristics.

**Variable**	**N**	**Previous radiotherapy**			**Previous extracranial radiotherapy**		
		**No (*n* = 318)**	**Yes (*n* = 324)**	***P*****-value**	**No (*n* = 354)**	**Yes (*n* = 288)**	***P*****-value**
**Sex**							
Male	360	168(53%)	192(59%)	0.101	198(56%)	162(56%)	0.936
Female	282	150(47%)	132(41%)		156(44%)	126(44%)	
**Age, years**							
≥55	312	144(45%)	168(52%)	0.497	174(49%)	138(48%)	0.755
<55	330	174(55%)	156(48%)		180(51%)	150(52%)	
**ECOG PS Score**							
0	122	60(19%)	62(19%)	0.992	70(20%)	52(18%)	0.859
1	412	204(64%)	208(64%)		225(63%)	187(65%)	
2	108	54(17%)	54(17%)		59(17%)	49(17%)	
**Histopathological classification**							
Squamous cell	198	90(28%)	108(33%)	0.168	120(34%)	78(27%)	0.063
Adenocarcinoma or other	444	228(72%)	216(67%)		234(66%)	210(73%)	
**Smoking history**							
Never-smoker	402	210(66%)	192(59%)	0.076	233(66%)	169(58%)	0.409
Former/current smoker	240	108(34%)	132(41%)		121(34%)	119(42%)	
**Diagnosis method**							
CT guided biopsy	264	126(40%)	138(43%)	0.001	138(39%)	126(44%)	0.001
Pleural effusion cytology	144	78(25%)	66(20%)		90(25%)	54(19%)	
Thoracotomy	174	72(23%)	102(31%)		78(22%)	96(33%)	
Neck lymph node biopsy	60	42(12%)	18(6%)		48(14%)	12(4%)	
**Color of pleural effusion**							
Bloody	426	222(70%)	204(63%)	0.066	246(69%)	180(63%)	0.062
Light yellow	216	96(30%)	120(37%)		108(31%)	108(37%)	
**Hematologic findings**							
Neutrophil count, mean ± IQR, × 10^3^/μl		6.12 ± 1.78	4.48 ± 1.34	0.032	6.09 ± 1.81	4.4 ± 1.32	0.034
Total lymphocyte count, mean ± IQR, × 10^3^/μl		1.34 ± 0.35	2.21 ± 0.70	0.021	1.35 ± 0.36	2.19 ± 0.69	0.022
Neutrophil-to-lymphocyte ratio		4.56 ± 1.36	2.06 ± 0.70	<0.01	4.52 ± 1.41	2.02 ± 0.75	<0.01
**Intrapleural chemotherapy before IL-2**							
Yes	528	258(81%)	270(83%)	0.466	286(81%)	242(84%)	0.286
No	114	60(19%)	54(17%)	68(19%)	46(19%)		
**History of brain metastases**			68(21%)		36(10%)	32(11%)	
**No. of previous systemic therapies, mean (range)**	2(0-5)	2(0-5)	0.017	2(0-5)	3(0-6)	0.021	
**Previous systemic therapies before IL-2**							
Yes	486	236(74%)	250(77%)	0.384	259(73%)	227(79%)	0.097
No	156	82(26%)	74(23%)		95(27%)	61(21%)	
**Radiotherapy schedule before distant metastasis**							
ChT → CCRT			140(43%)			140(49%)	
CCRT → ChT			61(19%)			61(21%)	
ChT → RT			66(20%)			66(23%)	
CCRT alone			21(7%)			21(7%)	
Intracranial radiotherapy			36(11%)			0	
**Radiotherapy technology**							
Conventional radiotherapy			164(%)			148(51%)	
3D-CRT/MRT			160(%)			140(49%)	
**Previous SABR**							
Yes	20		20(%)			20(7%)	
No	622		304(%)			268(93%)	

*ECOG, Eastern Cooperative Oncology Group; IMRT, intensity modulated radiotherapy; 3D-CRT, three dimensional conformal radiotherapy; SBRT, stereotactic body radiotherapy or stereotactic radiosurgery*.

Regardless of whether patients had received prior radiotherapy or not, significant differences were found between groups with regard to sex, age, ECOG status, histopathologic classification, smoking history, diagnosis method, color of MPE, and history of concurrent intrapleural chemotherapy (Table 1). Patients who had prior radiotherapy had significantly higher frequency of brain metastases and had received more systemic therapies than patients who had not had prior radiotherapy (Table 1). Frequency of brain metastases was similar among patients who had received extracranial radiotherapy and those who had not had prior extracranial radiotherapy.

### Survival Outcomes

**Figure 2:**
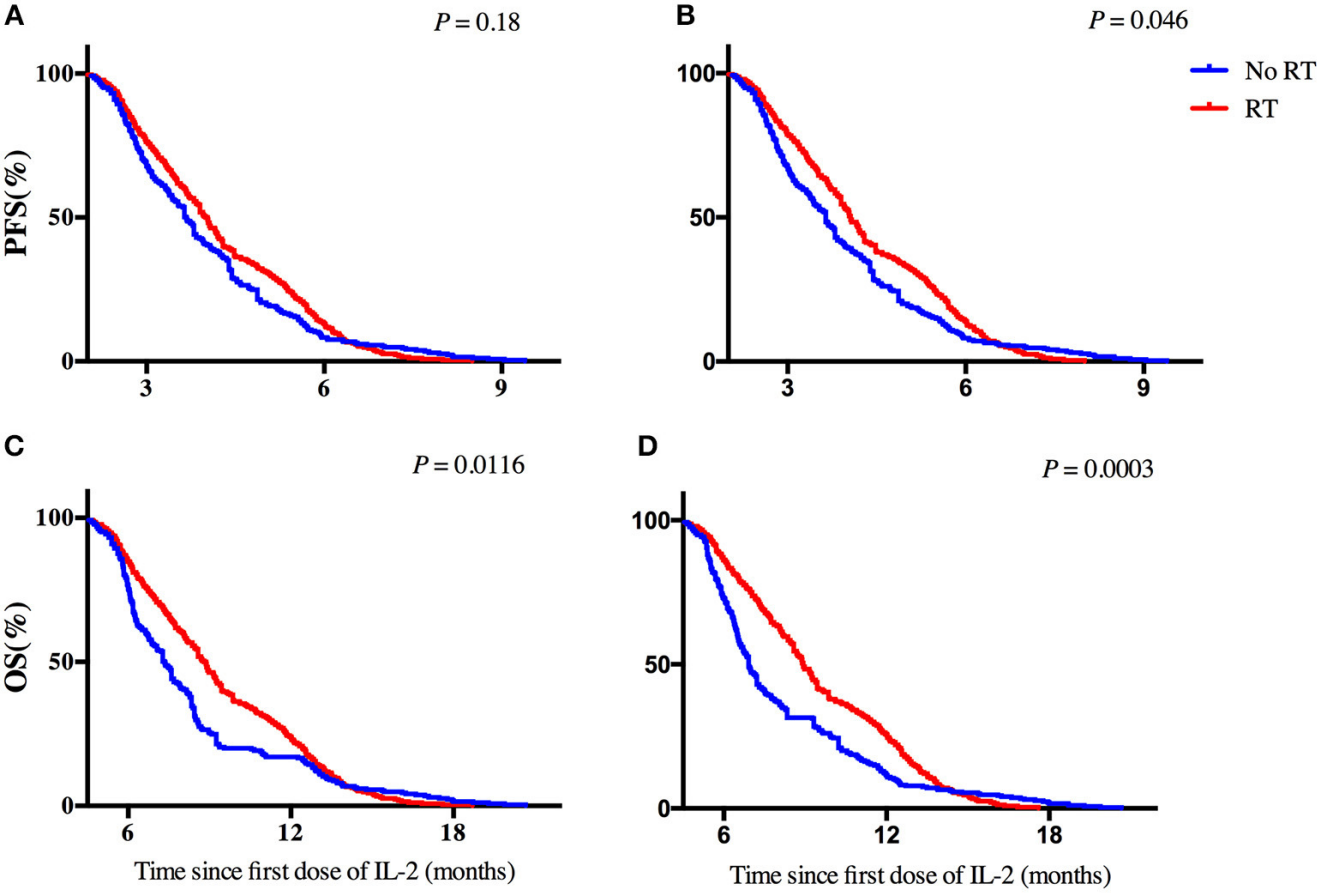
Effect of previous radiotherapy on progression-free survival and overall survival for patients with IL-2. **(A,B)** Progression-free survival in patients according to a history of **(A)** any radiotherapy or **(B)** extracranial radiotherapy. **(C,D)** Overall survival in patients according to a history of **(C)** any radiotherapy or **(D)** extracranial radiotherapy. Hazard ratios [HRs] are shown.

PFS time was similar among patients given IL-2 regardless of whether they had had prior radiotherapy or not (median PFS time 4.0 months prior vs. 3.67 months no prior, *p* = 0.18; Figure 2A). PFS time for patients who received IL-2 preceded by extracranial radiotherapy was longer than that for patients who had not had extracranial radiotherapy (median PFS time 4.06 months extra vs. 3.64 months no extra, *p* = 0.046; Figure 2B). Otherwise, previous radiotherapy or previous extracranial radiotherapy did not confer an advantage in PFS for patients with cisplatin (Supplementary Figure 1). In univariate analysis of the 642 patients who received intrapleural IL-2, having had any prior radiotherapy (*p* = 0.007) and having had extracranial radiotherapy (*p* = 0.003) were associated with longer PFS. Multivariate analysis revealed that having had any radiotherapy and extracranial radiotherapy were independent predictors of PFS (Table 2).

**Table 2:** Predictors associated with progression free survival (PFS).

	PFS^*^		**Any previous RT and PFS***^†^*			**Previous extracranial RT and PFS***^†^*		
	**Wald x2**	***p*** **value**	**HR**	**95% CI**	***p*** **value**	**HR**	**95% CI**	***p*** **value**
**Sex**	1.778	0.182						
(Male vs Female)								
**Age**	0.056	0.812						
(≥55 vs <55)								
**ECOG PS Score**	0.104	0.747						
(0 vs 1 vs 2)								
**Histopathological classification**	1.860	0.173						
(Adenocarcinoma and other vs Squamous)								
**Smoking history**	2.845	0.092	0.892	0.747–1.064	0.204	0.862	0.721–1.029	0.101
(Never vs Former/current)								
**Color**	0.178	0.673						
(Bloody vs Yellow)								
**Previous systematic therapy**	1.931	0.165						
(Yes vs No)								
**Previous intrapleural chemotherapy**	1.859	0.173						
(Yes vs No)								
**Any previous radiotherapy**	7.299	0.007	0.805	0.677–0.957	0.014			
(Yes vs No)								
**Previous extracranial radiotherapy**	9.048	0.003				0.752	0.632–0.895	0.001
(Yes vs No)								

*Progression-free survival was defined as the time from the first administration of I.P. IL-2 to disease progression or death*.

*HR, hazard ratio*.

* *Univariate analysis*.

† *Multivariate analysis*.

As for OS time, among the patients given IL-2, having received any radiotherapy was associated with longer OS time than no radiotherapy (median OS time 8.8 months vs. 7.34 months, *p* = 0.0116; Figure 2C), as was having received extracranial radiotherapy compared with no extracranial radiotherapy (median OS time 8.93 months vs. 6.92 months, *p* = 0.0003; Figure 2D). Otherwise, previous radiotherapy or previous extracranial radiotherapy did not confer any advantage in OS for patients with cisplatin (Supplementary Figure 1). In univariate analysis of the 642 patients who received intrapleural IL-2, having had any prior radiotherapy (*p* < 0.001) and extracranial radiotherapy (*p* < 0.001) were associated with longer OS. Multivariate analysis revealed that having had any radiotherapy and extracranial radiotherapy were independent predictors of OS (Table 3).

**Table 3:** Predictors associated with overall survival (OS).

	OS^*^		**Any previous RT and OS*^†^***			**Previous extracranial RT and OS*^†^***		
	**Wald x2**	***p*** **value**	**HR**	**95% CI**	***p*** **value**	**HR**	**95% CI**	***p*** **value**
**Sex**	1.610	0.205						
(Male vs. Female)								
**Age**	0.030	0.863						
(≥55 vs <55)								
**ECOG PS Score**	0.071	0.790						
(0 vs. 1 vs. 2)								
**Histopathological classification**	1.456	0.228						
(Adenocarcinoma and other vs. Squamous)								
**Smoking history**	3.747	0.053	0.884	0.740–1.054	0.169	0.875	0.733–1.046	0.142
(Never vs. Former/current)								
**Color**	0.269	0.604						
(Bloody vs. Yellow)								
**Previous systematic therapy**	1.994	0.158						
(Yes vs. No)								
**Previous intrapleural chemotherapy**	1.673	0.196						
(Yes vs. No)								
**Any previous radiotherapy**	15.033	<0.001	0.726	0.611–0.864	<0.001			
(Yes vs. No)								
**Previous extracranial radiotherapy**	17.101	<0.001				0.653	0.549–0.778	<0.001
(Yes vs. No)								

*Overall survival was defined as the time from the first dose of intrapleural interkeukin-2 until disease progression or death*.

*OS, overall survival*.

### Hematologic Outcomes

Patients who had had any previous radiotherapy had higher TLC than patients with no prior radiotherapy (1.34 ± 0.35 vs. 2.21 ± 0.70, *p* = 0.021); had lower neutrophil counts than patients with no prior radiotherapy (6.12 ± 1.78 vs. 4.48 ± 1.34, *p* = 0.032); and had lower NLR than patients with no prior radiotherapy (4.56 ± 1.36 vs. 2.06 ± 0.70, *p* < 0.01). These patterns also held for patients who had had extracranial radiotherapy vs. no extracranial radiotherapy (TLC: 6.09 ± 1.81 vs. 4.4 ± 1.32, *p* = 0.034; neutrophils: 1.35 ± 0.36 vs. 2.19 ± 0.69, *p* = 0.022); and NLR: 4.52 ± 1.41 vs. 2.02 ± 0.75, *p* < 0.01).

### Treatment Efficacy

Short-term treatment efficacy did not differ substantially between patients who had had any radiotherapy and those who had not had any radiotherapy. Treatment outcomes were also no different between patients who had extracranial radiotherapy vs. no extracranial radiotherapy (Table 4).

**Table 4:** Short-term treatment efficacy for patients with intrapleural IL-2.

	**Previous radiotherapy**			**Previous extracranial radiotherapy**		
	**No**	**Yes**	***P*** **Value**	**No**	**Yes**	***P*** **Value**
	**(*****n*** **= 318)**	**(*****n*** **= 324)**		**(*****n*** **= 354)**	**(*****n*** **= 288)**	
Complete response, no.	16	20	0.529	18	18	0.585
Partial response, no.	140	139	0.317	145	134	0.157
Stable disease, no.	60	52	0.347	63	49	0.797
Disease control rate	67.92	65.12	0.452	63.84	69.79	0.112
Objective response rate, %	49.06	49.07	0.669	46.05	52.78	0.0897

## Discussion

Preclinical and clinical studies have shown that radiotherapy can enhance antitumor immune responses. A secondary analysis of the KEYNOTE-001 phase 1 trial, in particular, suggested that previous radiotherapy may improve the outcomes of patients given pembrolizumab relative to those for patients not given radiotherapy. Here we sought to determine whether radiotherapy combined with another well-known immune agent would produce similar radio-memory effect. In our retrospective analysis of outcomes among patients given intrapleural IL-2 or cisplatin for MPE in light of prior receipt of radiotherapy for NSCLC, we found that having received radiotherapy before IL-2 led to longer PFS and OS times compared with patients who had not had prior radiotherapy. No such results were found for patients given cisplatin but not IL-2. We further found that patients who had had any prior radiotherapy or prior extracranial radiotherapy had higher TLC, lower neutrophil counts, and lower NLR than those who had no prior radiotherapy which generally are associated with better treatment efficacy and longer survival (16, 17). To our knowledge, this was the largest such analysis undertaken to date, and its results strongly suggest that having had radiotherapy may improve the efficacy of immune therapy other than checkpoint inhibitors. These findings are consistent with the results of several preclinical studies indicating significant, synergistic antitumor effects achieved by combining radiotherapy with immunotherapy. Our findings thus seem to demonstrate that combining radiotherapy with immunotherapy other than the previously studied anti-PD1/PDL1 can indeed induce a “radio-memory” effect.

IL-2, also known as T-cell growth factor, includes a range of bioactive cytokines produced primarily by activated CD4+ T cells and CD8+ T cells, acts as a growth factor for all T-cell subsets and can also promote the activation of B-cell proliferation (18, 19). IL-2 is also important in the regulation of the immune response, including antibody responses, hematopoiesis, and tumor surveillance. Numerous animal models have shown that combining radiotherapy with IL-2 has a synergistic antitumor effect (20–23) and has been shown to be useful clinically in oral cancer (24), renal cell carcinoma (25), and prostate cancer (26). Our results are consistent with these studies. In a study published in 2011, Koji suggested that radiotherapy combined with IL-2 may have radiopharmaceutical effects (27); however, to our knowledge, no studies to date have demonstrated a radio-memory effect.

In our study, patients given intrapleural IL-2 who had previously received radiotherapy for NSCLC had better OS than those who had not had radiotherapy (*p* = 0.0116, Figure 2C), but no differences were found in short-term efficacy or PFS between these to groups (*p* = 0.18, Figure 2A). We further found better PFS and OS among patients who had received extracranial irradiation than among those who had not received extracranial irradiation (*p* = 0.046, Figure 2B; *p* = 0.0003, Figure 2D). These findings may reflect the following. First, compared to extracranial radiotherapy, cranial radiotherapy will damage more lymphocyte cells and some believe that cranial irradiation cannot change the immune microenvironment in the brain (28). Second, the immune microenvironment within the brain may not change because of the presence of the blood-brain barrier. Even if such changes in the immune system did occur within the brain, it is unlikely that this effect would extend to the immune microenvironment throughout the body, as the blood-brain barrier serves as an obstacle.

Typically the choice of radiation dose, distribution, sequence, and target area is made to ensure thorough destruction of tumor cells while minimizing damage to surrounding normal tissues. When radiotherapy is used as an intervention for immunization, however, this classical approach may require adjustment. For example, it may not be necessary to include the entire tumor area to high-dose radiation, because exposing only a part of the tumor may be required to trigger the immune system. Radiation dose and segmentation schemes that trigger an immune response probably depend on many unknown factors, but the required dose is likely to be much lower than the dose required for definitive radiotherapy, which would in turn reduce the likelihood of adverse reactions. Finally, proton therapy may be more suitable than photon therapy for combining with immune therapy, as proton therapy doses are usually lower and protons may have an as-yet unrecognized role in immunotherapy. However, more comprehensive research in this area is required before recommendations can be made.

Our study of patients with NSCLC who also developed MPE involved a large number of patients with which to analyze the overall clinical effect of previous radiotherapy when those patients were given IL-2. Nevertheless, our study had several limitations, chief among them its retrospective nature, with the attendant unclear inclusion criteria and somewhat inaccurate statistical analyses. Second, the effects on the clinical endpoints studied (OS and PFS) were relatively modest, and the statistical significance levels were marginal. Third, our data could not shed light on the mechanism underlying the radio-memory effect of previous radiotherapy. Also, it is possible that bias may have been introduced by differences in standards of living among patients, perhaps including distance from and access to radiotherapy facilities. Overall, although this study could be considered hypothesis-generating; its conclusions should be validated through prospective controlled trials. From surgery to medicine, and from radiotherapy to immunotherapy, many approaches have been attempted to eradicate cancer. However, single treatments have had relatively modest success at best, and hence the greatest challenge faced by modern-day oncologists is to identify the optimal combinations of existing forms of cancer therapy.

## Conclusions

Our findings suggest that previous receipt of radiotherapy for NSCLC may enhance the efficacy of IL-2 for treating MPE and improve outcomes for such patients. They further suggest that the radio-memory effect is not restricted to combining radiotherapy with PD1/PDL1 inhibitors but may extend to other immunotherapy agents. Future clinical trials involving radiotherapy and immunotherapy should consider this observation in designing more rational regimens.

## Author Contributions

JmY and JbY designed the study. DC, XS, HW, WM, YW, YM, ZG, and SC participated in the collection and analysis the data. DC and XS wrote the manuscript. KL and JbY were responsible of the critical review and revision of this manuscript. All authors provided the approval of the final manuscript for submission.

### Conflict of Interest Statement

The authors declare that the research was conducted in the absence of any commercial or financial relationships that could be construed as a potential conflict of interest.

## Supplementary Material

Supplementary Figure 1 Effect of previous radiotherapy on progression-free survival and overall survival for patients with Cisplatin. **(A,B)** Progression-free survival in patients according to a history of **(A)** any radiotherapy or **(B)** extracranial radiotherapy. **(C,D)** Overall survival in patients according to a history of **(C)** any radiotherapy or **(D)** extracranial radiotherapy. Hazard ratios [HRs] are shown.

## Abbreviations

NSCLC: non-small cell lung cancer
IL-2: interleukin-2
MPE: malignant pleural effusion
ECOG: Eastern Cooperative Oncology Group
SBRT: stereotactic ablative radiotherapy or radiosurgery
CR: complete response
PR: partial response
SD: stable disease
DCR: disease control rate
ORR: objective response rate.

## References

[1] Weichselbaum RR Liang H Deng L Fu YX. Radiotherapy and immunotherapy: a beneficial liaison? Nat Rev Clin Oncol. (2017) 14:365–79. 10.1038/nrclinonc.2016.211 28094262

[2] Wersäll PJ Blomgren H Pisa P Lax I Kälkner KM Svedman C. Regression of non-irradiated metastases after extracranial stereotactic radiotherapy in metastatic renal cell carcinoma. Acta Oncol. (2006) 45:493–7. 10.1080/02841860600604611 16760190

[3] Herrera FG Bourhis J Coukos G. Radiotherapy combination opportunities leveraging immunity for the next oncology practice. CA Cancer J Clin. (2017) 67:65–85. 10.3322/caac.21358 27570942

[4] Mole RH. Whole body irradiation; radiobiology or medicine? Br J Radiol. (1953) 26:234–41. 13042090 10.1259/0007-1285-26-305-234

[5] Ng J Dai T. Radiation therapy and the abscopal effect: a concept comes of age. Ann Transl Med. (2016) 4:118. 10.21037/atm.2016.01.32 27127771 PMC4828732

[6] Levy A Chargari C Marabelle A Perfettini JL Magné N Deutsch E. Can immunostimulatory agents enhance the abscopal effect of radiotherapy? Eur J Cancer (2016) 62:36–43. 10.1016/j.ejca.2016.03.067 27200491

[7] Van de Walle M Demol J Staelens L Rottey S. Abscopal effect in metastatic renal cell carcinoma. Acta Clin Belg. (2017) 72:245–9. 10.1080/17843286.2016.1201614 27425038

[8] Reynders K Illidge T Siva S Chang JY De Ruysscher D. The abscopal effect of local radiotherapy: using immunotherapy to make a rare event clinically relevant. Cancer Treat Rev. (2015) 41:503–10. 10.1016/j.ctrv.2015.03.011 25872878 PMC4816218

[9] Frey B Rubner Y Wunderlich R Weiss EM Pockley AG Fietkau R. Induction of abscopal anti-tumor immunity and immunogenic tumor cell death by ionizing irradiation-implications for cancer therapies. Curr Med Chem. (2012) 19:1751–64. 10.2174/092986712800099811 22414083

[10] Kanegasaki S Matsushima K Shiraishi K Nakagawa K Tsuchiya T. Macrophage inflammatory protein derivative ECI301 enhances the alarmin-associated abscopal benefits of tumor radiotherapy. Cancer Res. (2014) 74:5070–8. 10.1158/0008-5472.CAN-14-0551 25038226

[11] Marincola FM Jaffee EM Hicklin DJ Ferrone S. Escape of human solid tumors from T-cell recognition: molecular mechanisms and functional significance. Adv Immunol. (2000) 74:181–273. 10.1016/S0065-2776(08)60911-6 10605607

[12] Zeng J See AP Phallen J Jackson CM Belcaid Z Ruzevick J. Anti-PD-1 blockade and stereotactic radiation produce long-term survival in mice with intracranial gliomas. Int J Radiat Oncol Biol Phys. (2013) 86:343–49. 10.1016/j.ijrobp.2012.12.025 23462419 PMC3963403

[13] Demaria S Coleman CN Formenti SC. Radiotherapy: changing the game in immunotherapy. Trends Cancer (2016) 2:286–94. 10.1016/j.trecan.2016.05.002 27774519 PMC5070800

[14] Shaverdian N Lisberg AE Bornazyan K Veruttipong D Goldman JW Formenti SC. Previous radiotherapy and the clinical activity and toxicity of pembrolizumab in the treatment of non-small-cell lung cancer: a secondary analysis of the KEYNOTE-001 phase 1 trial. Lancet Oncol. (2017) 18:895–903. 10.1016/S1470-2045(17)30380-7 28551359 PMC5538772

[15] Antonia SJ Villegas A Daniel D Vicente D Murakami S Hui R. Durvalumab after chemotherapy in stage III non-small cell lung cancer. N Engl J Med. (2017) 377:1919–29. 10.1056/NEJMoa1709937 28885881

[16] Guthrie GJ Charles KA Roxburgh CS Horgan PG McMillan DC Clarke SJ. The systemic inflammation-based neutrophil-lymphocyte ratio: experience in patients with cancer. Crit Rev Oncol Hematol. (2013) 88:218–30. 10.1016/j.critrevonc.2013.03.010 23602134

[17] Miyamoto R Inagawa S Sano N Tadano S Adachi S Yamamoto M. The neutrophil-to-lymphocyte ratio (NLR) predicts short-term and long-term outcomes in gastric cancer patients. Eur J Surg Oncol. (2018) 44:607–12. 10.1016/j.ejso.2018.02.003 29478743

[18] Ross SH Cantrell DA. Signaling and function of interleukin-2 in T lymphocytes. Annu Rev Immunol. (2018) 36:411–33. 10.1146/annurev-immunol-042617-053352 29677473 PMC6472684

[19] Berraondo P Sanmamed MF Ochoa MC Etxeberria I Aznar MA Pérez-Gracia JL. Cytokines in clinical cancer immunotherapy. Br J Cancer (2018). [Epub ahead of print]. 10.1038/s41416-018-0328-y 30413827 PMC6325155

[20] Dybal EJ Haas GP Maughan RL Sud S Pontes JE Hillman GG. Synergy of radiation therapy and immunotherapy in murine renal cell carcinoma. J Urol. (1992) 148:1331–7. 10.1016/S0022-5347(17)36903-3 1404669

[21] Younes E Haas GP Dezso B Ali E Maughan RL Kukuruga MA. Local tumor irradiation augments the response to IL-2 therapy in a murine renal adenocarcinoma. Cell Immunol. (1995) 165:243–51. 10.1006/cimm.1995.1211 7553889

[22] Dezso B Haas GP Hamzavi F Kim S Montecillo EJ Benson PD. The mechanism of local tumor irradiation combined with interleukin 2 therapy in murine renal carcinoma: histological evaluation of pulmonary metastases. Clin Cancer Res. (1996) 2:1543–52. 9816331

[23] Jürgenliemk-Schulz IM Renes IB Rutgers DH Everse LA Bernsen MR Den Otter W. Anti-tumor effects of local irradiation in combination with peritumoral administration of low doses of recombinant interleukin-2 (rIL-2). Radiat Oncol Investig. (1997) 5:54–61. 9303058 10.1002/(SICI)1520-6823(1997)5:2<54::AID-ROI3>3.0.CO;2-I

[24] Tímár J Forster-Horváth C Lukits J Döme B Ladányi A Remenár E. The effect of leukocyte interleukin injection (Multikine) treatment on the peritumoral and intratumoral subpopulation of mononuclear cells and on tumor epithelia: a possible new approach to augmenting sensitivity to radiation therapy and chemotherapy in oral cancer – a multicenter phase I / II clinical trial. Laryngoscope (2003) 113:2206–17. 10.1097/00005537-200312000-00031 14660929

[25] Redman BG Hillman GG Flaherty L Forman J Dezso B Haas GP. Phase II trial of sequential radiation and interleukin 2 in the treatment of patients with metastatic renal cell carcinoma. Clin Cancer Res. (1998) 4:283–6. 9516912

[26] Lechleider RJ Arlen PM Tsang KY Steinberg SM Yokokawa J Cereda V. Safety and immunologic response of a viral vaccine to prostate-specific antigen in combination with radiation therapy when metronomic-dose interleukin 2 is used as an adjuvant. Clin Cancer Res. (2008) 14:5284–91. 10.1158/1078-0432.CCR-07-5162 18698048 PMC2639763

[27] Yasuda K Nirei T Tsuno NH Nagawa H Kitayama J. Intratumoral injection of interleukin-2 augments the local and abscopal effects of radiotherapy in murine rectal cancer. Cancer Sci. (2011) 102:1257–63. 10.1111/j.1349-7006.2011.01940.x 21443690

[28] Yovino S Kleinberg L Grossman SA Narayanan M Ford E. The etiology of treatment-related lymphopenia in patients with malignant gliomas: modeling radiation dose to circulating lymphocytes explains clinical observations and suggests methods of modifying the impact of radiation on immune cells. Cancer Invest. (2013) 31:140–4. 10.3109/07357907.2012.762780 23362951 PMC3991115

