# Corrigendum: Previous Radiotherapy Increases the Efficacy of IL-2 in Malignant Pleural Effusion: Potential Evidence of a Radio-Memory Effect?

**DOI:** 10.3389/fimmu.2021.649620

**Published:** 2021-03-23

**Authors:** Dawei Chen, Xinyu Song, Haiyong Wang, Zhenwu Gao, Wenjuan Meng, Shuquan Chen, Yunfeng Ma, Youda Wang, Kong Li, Jinming Yu, Jinbo Yue

**Affiliations:** ^1^ Department of Radiation Oncology, Shandong Cancer Hospital affiliated to Shandong University, Jinan, China; ^2^ Department of Internal Medicine-Oncology, Shandong Cancer Hospital affiliated to Shandong University, Jinan, China; ^3^ School of Medicine and Life Sciences, University of Jinan-Shandong Academy of Medical Sciences, Jinan, China; ^4^ Department of oncology, Affiliated Hospital of Weifang Medical University, Weifang, China; ^5^ Weifang People’s Hospital, Weifang, China; ^6^ Laiwu Hospital of Traditional Chinese Medicine, Laiwu, China; ^7^ Laiwu People’s Hospital, Laiwu, China; ^8^ Linyi City People’s Hospital, Linyi, China

**Keywords:** non-small-cell lung cancer (NSCLC), radiotherapy, radio-memory effect, immunotherapy, interleukin-2 (IL-2), malignant pleural effusion (MPE)

In the original article, there were mistakes in **Figure 1** and **Tables 1**–**3** as published.

**Figure 1:**
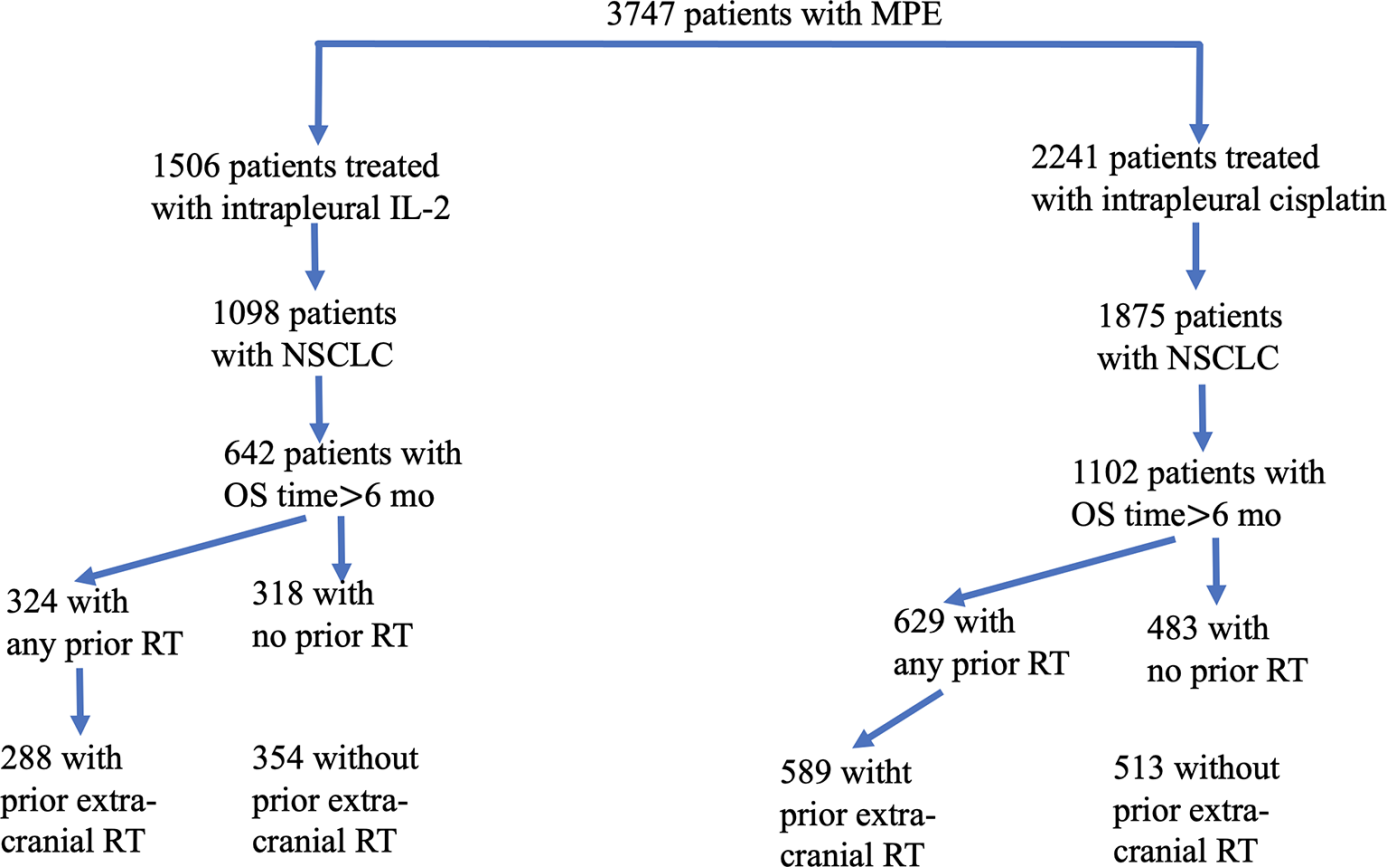
Flowchart of eligible patients enrolled in this study. From a total of 3,747 patients with malignant pleural effusion (MPE), we identified 1,506 who had been treated with interpleural interleukin-2 (IL-2) and 2,241 who had been treated with intrapleural cisplatin. Of the 1,098 patients given IL-2 (and the 1,875 patients given cisplatin) who had non-small cell lung cancer, 642 who had received IL-2 survived for more than 6 months, and 1,102 who had received cisplatin survived for more than 6 months. Patients in each group were subdivided according to whether they had any vs. no radiotherapy (RT), or extracranial vs. no extracranial RT.

**Table 1:** Baseline characteristics.

Variable	Previous radiotherapy				Previous extracranial radiotherapy		
	N	No (n=318)	Yes (n=324)	p value	No (n=354)	Yes (n=288)	p value
**Sex**							
Male	360	168(53%)	192(59%)	0.101	198(56%)	162(56%)	0.936
Female	282	150(47%)	132(41%)		156(44%)	126(44%)	
**Age, years**							
≥55	312	144(45%)	168(52%)	0.497	174(49%)	138(48%)	0.755
<55	330	174(55%)	156(48%)		180(51%)	150(52%)	
**ECOG PS Score**							
0	122	60(19%)	62(19%)	0.992	70(20%)	52(18%)	0.859
1	412	204(64%)	208(64%)		225(63%)	187(65%)	
2	108	54(17%)	54(17%)		59(17%)	49(17%)	
**Histopathological classification**							
Squamous cell	198	90(28%)	108(33%)	0.168	120(34%)	78(27%)	0.063
Adenocarcinoma or other	444	228(72%)	216(67%)		234(66%)	210(73%)	
**Smoking history**							
Never-smoker	402	210(66%)	192(59%)	0.076	233(66%)	169(58%)	0.409
Former/current smoker	240	108(34%)	132(41%)		121(34%)	119(42%)	
**Diagnosis method**							
CT guided biopsy	264	126(40%)	138(43%)	0.001	138(39%)	126(44%)	0.001
Pleural effusion cytology	144	78(25%)	66(20%)		90(25%)	54(19%)	
Thoracotomy	174	72(23%)	102(31%)		78(22%)	96(33%)	
Neck lymph node biopsy	60	42(12%)	18(6%)		48(14%)	12(4%)	
**Color of Pleural Effusion**							
Bloody	426	222(70%)	204(63%)	0.066	246(69%)	180(63%)	0.062
Light yellow	216	96(30%)	120(37%)		108(31%)	108(37%)	
**Hematologic Findings**							
Neutrophil count, mean ± IQR, × 10^3^/μl		6.12 ± 1.78	4.48 ± 1.34	0.032	6.09 ± 1.81	4.4 ± 1.32	0.034
Total lymphocyte count, mean ± IQR, × 10^3^/μl		1.34 ± 0.35	2.21 ± 0.70	0.021	1.35 ± 0.36	2.19 ± 0.69	0.022
Neutrophil-to-lymphocyte ratio		4.56 ± 1.36	2.06 ± 0.70	<0.01	4.52 ± 1.41	2.02 ± 0.75	<0.01
**Intrapleural chemotherapy chemotherapy before IL-2**							
Yes	528	258(81%)	270(83%)	0.466	286(81%)	242(84%)	0.286
No	114	60(19%)	54(17%)		68(19%)	46(19%)	
**History of brain metastases**			68(21%)		36(10%)	32(11%)	
**No. Of previous systemic therapies, mean (range)**		2(0-5)	2(0-5)	0.017	2(0-5)	3(0-6)	0.021
**Previous systemic therapies before IL-2**							
Yes	486	236(74%)	250(77%)	0.384	259(73%)	227(79%)	0.097
No	156	82(26%)	74(23%)		95(27%)	61(21%)	
**Radiotherapy schedule before distant metastasis**							
ChT→CCRT			140(43%)			140(49%)	
CCRT→ChT			61(19%)			61(21%)	
ChT→RT			66(20%)			66(23%)	
CCRT alone			21(7%)			21(7%)	
Intracranial radiotherapy			36(11%)			0	
**Radiotherapy technology**							
Conventional radiotherapy			164(%)			148(51%)	
3D-CRT/MRT			160(%)			140(49%)	
**Previous SABR**							
Yes	20		20(%)			20(7%)	
No	622		304(%)			268(93%)	

ECOG, Eastern Cooperative Oncology Group; IMRT, intensity modulated radiotherapy; 3D-CRT, three dimensional conformal radiotherapy; SBRT, stereotactic body radiotherapy or stereotactic radiosurgery.

**Table 2:** Predictors associated with progression free survival (PFS).

	PFS*		Any previous RT and PFS†			Previous extracranial RT and PFS†		
	Wald x2	p value	HR	95% CI	p value	HR	95% CI	p value
**Sex** (Male vs Female)	1.778	0.182						
**Age** (≥55 vs <55)	0.056	0.812						
**ECOG PS Score** (0 vs 1 vs 2)	0.104	0.747						
**Histopathological classification** (Adenocarcinoma and other vs Squamous)	1.860	0.173						
**Smoking history** (Never vs Former/current)	2.845	0.092	0.892	0.747-1.064	0.204	0.862	0.721-1.029	0.101
**Colour** (Bloody vs Yellow)	0.178	0.673						
**Previous systematic therapy** (Yes vs No)	1.931	0.165						
**Previous intrapleural chemotherapy** (Yes vs No)	1.859	0.173						
**Any previous radiotherapy** (Yes vs No)	7.299	0.007	0.805	0.677-0.957	0.014			
**Previous extracranial radiotherapy** (Yes vs No)	9.048	0.003				0.752	0.632-0.895	0.001

Progression-free survival was defined as the time from the first administration of I.P. IL-2 to disease progression or death. HR, hazard ratio. *Univariate analysis.

^†^Multivariate analysis.

**Table 3:** Predictors associated with overall survival (OS)

	OS*		Any previous RT and OS†			Previous extracranial RT and OS†		
	Wald x2	p value	HR	95% CI	p value	HR	95% CI	p value
**Sex** (Male vs Female)	1.610	0.205						
**Age** (≥55 vs <55)	0.030	0.863						
**ECOG PS Score** (0 vs 1 vs 2)	0.071	0.790						
**Histopathological classification** (Adenocarcinoma and other vs Squamous)	1.456	0.228						
**Smoking history** (Never vs Former/current)	3.747	0.053	0.884	0.740-1.054	0.169	0.875	0.733-1.046	0.142
**Colour** (Bloody vs Yellow)	0.269	0.604						
**Previous systematic therapy** (Yes vs No)	1.994	0.158						
**Previous intrapleural chemotherapy** (Yes vs No)	1.673	0.196						
**Any previous radiotherapy** (Yes vs No)	15.033	<0.001	0.726	0.611-0.864	<0.001			
**Previous extracranial radiotherapy** (Yes vs No)	17.101	<0.001				0.653	0.549-0.778	<0.001

Overall survival was defined as the time from the first dose of intrapleural interkeukin-2 until disease progression or death. OS, overall survival.

In **Figure 1**, instead of “354 with any prior RT”, “288 with no prior RT”, “324 with prior extra-cranial RT” and “318 without prior extra-cranial RT”, it should be “324 with any prior RT”, “318 with no prior RT”, “288 with prior extra-cranial RT” and “354 without prior extra-cranial RT”, respectively.

Meanwhile, there were mistakes in **Table 1** due to some incorrect statistical results. Consequently, **Tables 2** and **3** also need to be revised as, after rechecking the data, ECOG score is not significant in **Table 2** These errors were caused by the carelessness and mis-operation in statistics and have been identified by the authors so that this would not happen in the future. The corrected **Figure 1** and **Table 1** to **3** appear below.

Consequently, a correction has been made to “RESULTS”, “Survival Outcomes”: paragraphs 1 and 2:

“In univariate analysis of the 642 patients who received intrapleural IL-2, having had any prior radiotherapy (p = 0.007) and having had extracranial radiotherapy (p = 0.003) were associated with longer PFS. Multivariate analysis revealed that having had any radiotherapy and extracranial radiotherapy were independent predictors of PFS (**Table 2**).”

“In univariate analysis of the 642 patients who received intrapleural IL-2, having had any prior radiotherapy (p < 0.001) and extracranial radiotherapy (p < 0.001) were associated with longer OS. Multivariate analysis revealed that having had any radiotherapy and extracranial radiotherapy were independent predictors of OS (**Table 3**).”

The authors apologize for this error and state that this does not change the scientific conclusions of the article in any way. The original article has been updated.

