# Supplementary figures and images for: Previous Radiotherapy Increases the Efficacy of IL-2 in Malignant Pleural Effusion: Potential Evidence of a Radio-Memory Effect?
Source: Front Immunol. 2018 Dec 11;9:2916. doi: 10.3389/fimmu.2018.02916 (PMC6297715; doi:10.3389/fimmu.2018.02916)

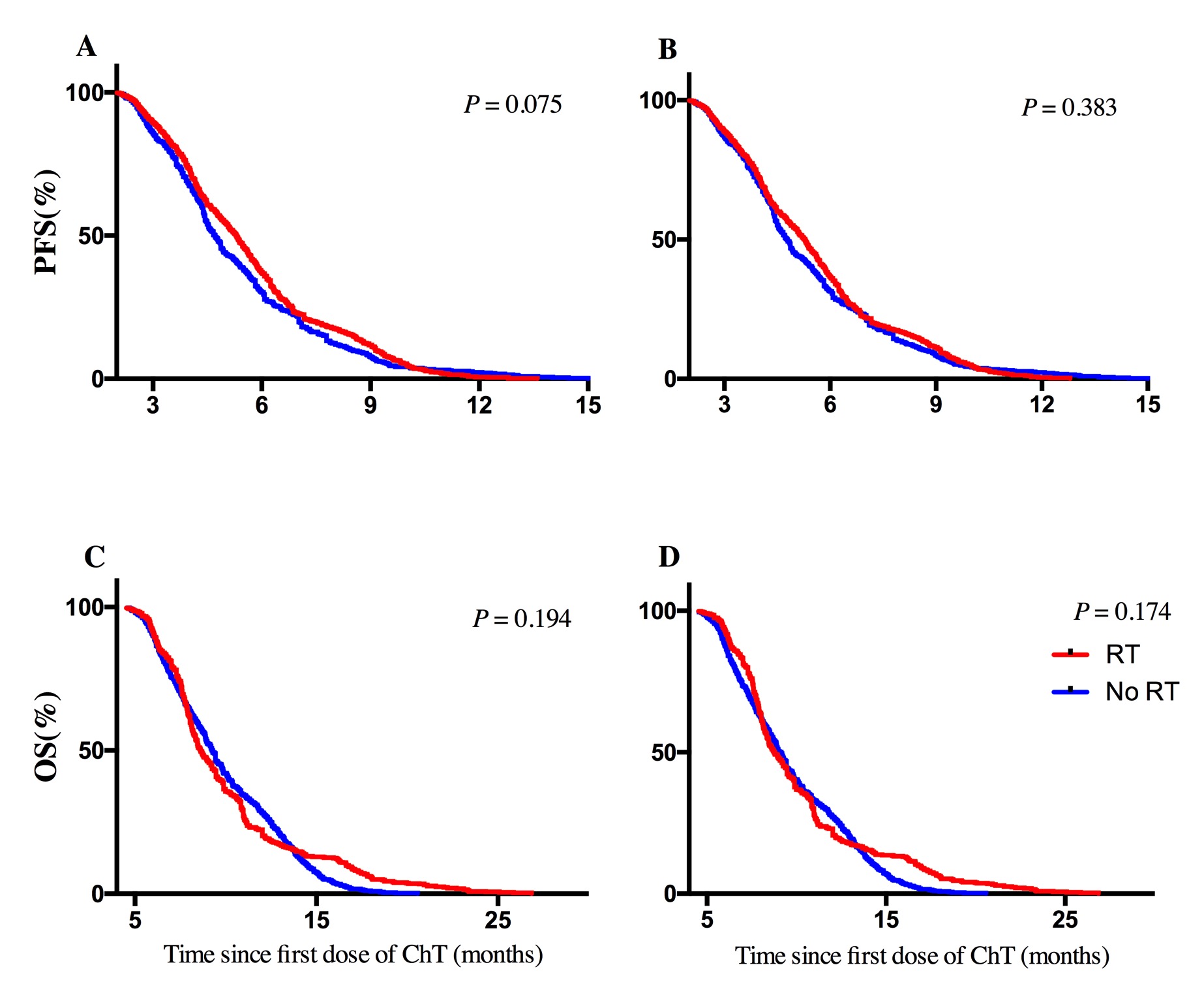

Supplement: Supplementary Figure 1 — Effect of previous radiotherapy on progression-free survival and overall survival for patients with Cisplatin. (A,B) Progression-free survival in patients according to a history of (A) any radiotherapy or (B) extracranial radiotherapy. (C,D) Overall survival in patients according to a history of (C) any radiotherapy or (D) extracranial radiotherapy. Hazard ratios [HRs] are shown. [file Image_1.JPEG]
